# Formulation and Optimization of Butenafine-Loaded Topical Nano Lipid Carrier-Based Gel: Characterization, Irritation Study, and Anti-Fungal Activity

**DOI:** 10.3390/pharmaceutics13071087

**Published:** 2021-07-16

**Authors:** Wael A. Mahdi, Sarah I. Bukhari, Syed Sarim Imam, Sultan Alshehri, Ameeduzzafar Zafar, Mohd Yasir

**Affiliations:** 1 Department of Pharmaceutics, College of Pharmacy, King Saud University, Riyadh 11451, Saudi Arabia; wmahdi@ksu.edu.sa (W.A.M.); sbukhari@ksu.edu.sa (S.I.B.); simam@ksu.edu.sa (S.S.I.); salshehri1@ksu.edu.sa (S.A.); 2 Department of Pharmaceutics, College of Pharmacy, Jouf University, Sakaka 72341, Saudi Arabia; zzafarpharmacian@gmail.com; 3 Department of Pharmacy, College of Health Sciences, Arsi University, Asella P.O. Box 396, Ethiopia

**Keywords:** butenafine, NLCs, topical gel, anti-fungal, optimization, irritation study

## Abstract

The present study aims to prepare and optimize butenafine hydrochloride NLCs formulation using solid and liquid lipid. The optimized selected BF-NLCopt was further converted into Carbopol-based gel for topical application for the treatment of fungal infection. Box Behnken design was employed to optimize the nanostructure lipids carriers (NLCs) using the lipid content (A), Tween 80 (B), and homogenization cycle (C) as formulation factors at three levels. Their effects were observed on the particle size (Y_1_) and entrapment efficiency (Y_2_). The selected formulation was converted into gel and further assessed for gel characterization, drug release, anti-fungal study, irritation study, and stability study. The solid lipid (Compritol 888 ATO), liquid lipid (Labrasol), and surfactant (tween 80) were selected based on maximum solubility. The optimization result showed a particle size of 111 nm with high entrapment efficiency of 86.35% for BF-NLCopt. The optimized BF-NLCopt converted to gel (1% *w*/*v*, Carbopol 934) and showed ideal gel evaluation results (drug content 99.45 ± 2.11, pH 6.5 ± 0.2, viscosity 519 ± 1.43 CPs). The drug release study result depicted a prolonged drug release (65.09 ± 4.37%) with high drug permeation 641.37 ± 46.59 µg (32.07 ± 2.32%) than BF conventional gel. The low value of irritation score (0.17) exhibited negligible irritation on the skin after application. The anti-fungal result showed greater efficacy than the BF gel at both time points. The overall conclusion of the results revealed NLCs-based gel of BF as an ideal delivery system to treat the fungal infection.

## 1. Introduction

Butenafine hydrochloride (BF) is a novel synthetic antifungal drug used in the treatment of different antifungal diseases. Chemically it is a benzylamine and naphthalene having the molecular formula C_23_H_27_N·HCl and molecular weight of 353.93 gm/mol [1]. It comes under a broad-spectrum antifungal agent and acts by inhibiting fungal squalene epoxidase synthesis of ergosterol, an important intermediate of fungal cell membrane synthesis [2,3]. BF has shown better therapeutic efficacy than terbinafine [4]. It is not used by the oral route as highly metabolized by the liver (1.5–3% oral bioavailability) and excreted in the urine.

Many different types of fungal germs live in the soil and other places in the environment. They can grow and multiply on the body surface and cause infection to the mouth, nails, and skin and produce fungal infection. The topical application of nanocarriers has shown a potential delivery system for fungal infection treatment. These nanocarriers have also been used in the topical treatment and possess superior therapeutic efficacy as compared to conventional cream and gel. These delivery systems improve the therapeutic efficacy by a different mechanism including enhancing permeation through subcutaneous, enhanced solubility, sustained drug release, and targeting potential to macrophage, and deeper skin layer [5,6]. Nano lipid carrier (NLC) is a second-generation lipid formulation prepared with a blend of solid lipid and liquid lipid [7]. They are prepared with physiological solid and liquid lipid, which is completely biodegradable. The used lipids for the NLCs preparation resemble the skin and sebum making them biocompatible with limited risk [8]. It possesses a lower melting point due to the presence of liquid lipid, maintains the particulate character, and remains solid at room temperature [9]. The presence of solid lipid in the NLCs helps to provide a prolonged drug-release pattern and also reduces systemic absorption. After application to the skin, it gives occlusive property [10,11]. The drug loading and entrapment are higher than the solid lipid nanoparticles due to the greater drug solubility in both solid and liquid lipid [12]. The nanoparticle size gives close contact to the stratum corneum and enhances the amount of drug permeation into the skin. Recently, NLCs are gaining popularity in the field of cosmetic, pharmaceutical, and food-delivery systems with a great potential for commercialization in the future [13].

The gel formulation is a semisolid dispersion system made up of inorganic and organic molecules enclosing and interpenetrated by a liquid. The inorganic particles give a three-dimensional structure [1,14]. The gel system should be washable, non-staining, stable at room temperature, and should not affect the biological nature of the drug [15]. There are numerous NLCs-based topical systems that have been reported to enhance the therapeutic efficacy of poorly soluble drugs like valdecoxib [11], luliconazole [16,17], oxiconazole [18], amphotericin B [7], itraconazole [19].

The design of the present study was divided into two steps. In the first step, BF-NLCs were prepared by the homogenization method. The formulation was optimized by the Box Behnken design (Design of expert, version 8.0.06, State-Ease Inc., Minneapolis, MN, USA) approach using independent variables as total lipid (A), tween 80 (B), and homogenization cycle (C) as independent variables. Their effects were observed on the particle size (Y_1_) and entrapment efficiency (Y_2_). In the second step, the optimized selected formulation (BF-NLCopt) was converted into a topical gel system using Carbopol as gelling agent to enhance the skin adhesion (BF-NLCopt gel). Carbopol offers excellent biocompatibility with the nano-drug delivery carriers, good gelling properties (easily swells at a small concentration of 1–2% *w*/*v*) with the ease of spreadability, thermal stability, and optimum rheological properties. The concentrations between 1% and 2% gave good consistency for the gel and it is generally recognized as a safe (GRAS) property. It also helps in controlling the release of the drug from the gel matrix. The prepared gel system was further assessed for gel characterization, drug release, permeation study, irritation study, and antifungal study.

## 2. Materials and Methods

### 2.1. Materials

BF was procured from Sigma Aldrich (St. Louis, MO, USA). The used lipids Preciol ATO 5, Compritol 888 ATO, and Labrasol were procured from Gattefosse Ltd. (Mumbai, India). Glyceryl monostearate, beeswax, stearic acid were obtained from SD fine Ltd. (Mumbai, India). Castor oil, almond oil, sunflower oil, and olive oil were obtained from a local market, Colaba (South Mumbai India). Cremophore EL, tween 20, tween 80, span 80, poloxamer 188, poloxamer F127 and span 20, methanol, HPLC grade water were procured from Sigma Aldrich (USA). Buffering reagents were obtained from the Central Drug House (Delhi, India).

### 2.2. Methods

#### 2.2.1. Screening of Solid and Liquid Lipid

The screening of lipid was performed to select the best lipid (solid and liquid) to formulate NLCs. The selection was performed on the criteria of the highest solubility of a drug into a lipid. The appropriate quantity (2 mg) of each solid lipid (Precirol ATO 5, Compritol 888 ATO, glyceryl monostearate, stearic acid, beeswax) was taken in glass vials and melted above 5 °C of the melting point. An excess amount of BF was added to the melted lipid. Similarly, the liquid lipids (castor oil, sunflower oil, labrasol, cremophore EL, almond oil, olive oil) were taken in glass vials. An excess of BF was added to the liquid lipid (2 mL). Both the lipids solid and liquid were vortexed (5 min) for complete solubilization and was allowed to stand for 72 h in an orbital shaker (Thermo-fisher Scientific, Mumbai, India). The mixture was centrifuged at 6000 rpm for 30 min and the supernatant separated. The supernatant was appropriately diluted and the concentration of BF dissolved in lipids was measured by a UV-spectrophotometer (Shimadzu-1800, Kyoto, Japan).

#### 2.2.2. Selection of Surfactant

The selection of surfactant for NLCs formulation was determined by the criteria of maximum solubility of BF in tested surfactants (Tween 20, Tween 80, Span 20, Span 80, Poloxamer, F127, Poloxamer F188). The appropriate volume of surfactant (2 mL) was taken in a glass vial and an excess amount of BF was added. The samples were and vortexed for 5 min and kept in an orbital shaker for 72 h. After that, the supernatant was centrifuged at 6000 rpm for 30 min. The supernatant was appropriately diluted and BF content in each surfactant was measured by a UV-spectrophotometer after dilution.

#### 2.2.3. Solid and Liquid Lipid Miscibility

The melted solid and liquid lipid in different compositions (9:1, 8:2, 7:3, 6:4, 5:5, 4:6) were mixed in a glass vial and vortexed. The mixture was kept aside at room temperature. One drop of the mixture was added to the filter paper, and the presence of an oil droplet was checked. The ratio of solid and liquid lipids at which they did not separate and exhibiting better miscibility (showing a single-phase system) was considered for further study.

#### 2.2.4. Optimization

From the preliminary screening study, parameters like lipid concentration, surfactant concentration, and homogenization cycle were chosen as pre-optimized parameters [19,20]. The optimization was done by design expert software (version 8.0.06, State-Ease Inc., Minneapolis, MN, USA) using the Box–Behnken design (3 factors at 3 levels). The three independent variables, lipid concentration (%, A), surfactant concentration (%, B), and homogenization cycle (number, C) were set at three levels i.e., low (−), medium (0), and high (+), as shown in Table 1. The effect of these three independent parameters was observed on two dependent variables, particle size (nm, Y_1_) and entrapment efficiency (%, Y_2_). The software showed a total of 17 compositions (5 commons) after adding the lower and higher values of lipid, surfactant, and homogenization cycle in the design expert software as shown in Table 2. The design depicted the effect of the independent parameters individually (A or B or C), their interaction effect (AB, AC, BC), and quadratic effect (A^2^, B^2^, and C^2^) on the particle size and entrapment efficiency. The outcome of the design was obtained in the form of a polynomial equation, and three-dimensional response surface graphs (3D and contour) [21,22].

#### 2.2.5. Formulation of BF-NLCs

BF-NLCs were prepared according to the reported method by Muller et al., 2000 with slight modification [23]. Lipid was melted around 5 °C above its melting point and the weighed amount of BF was added to the melted lipid. Separately, an aqueous surfactant solution was prepared and the same temperature was maintained as lipid. The hot surfactant solution was poured into the melted lipid phase with continuous stirring at 12,000 rpm using a magnetic stirrer for 30 min. The primary emulsion was formed and converted to the NLCs dispersion using a high-pressure homogenizer (Stansted Fluid Ltd., Harlo, UK) as shown in Table 2. The prepared NLCs dispersion was cooled down at room temperature and then lyophilized using mannitol (5% *w*/*v*) as a cryoprotectant.

#### 2.2.6. BF-NLCs Characterization

The particle size (PS), polydispersity index (PDI), zeta potential (ZP)of the prepared BF-NLCs were evaluated by a particle size analyzer (Nano-sizer, Malvern, UK). The sample was diluted 100-fold with Mili-Q water and the particle size, PDI, and ZP were measured. The morphology of the selected sample was assessed by transmission electron microscopy (TEM-TECNAI-G2, FEI, Eindhoven, The Netherlands). One drop of diluted sample was placed over a copper grid, and phosphotungstic acid dye was added to stain the sample. The grid was placed in an instrument and the image was captured.

#### 2.2.7. Entrapment and Loading Efficiency

EE and DL of BF in BF-NLCs were analyzed to check the amount of BF loaded and entrapped in the prepared NLCs. BF-NLCs were filled in a centrifugation tube and centrifuged at 15,000 rpm for 30 min (cooling centrifuge, Remi Lab, Mumbai, India). The supernatant was separated and diluted further to evaluate the unentrapped amount of drug in NLCs by measuring the absorbance with the help of a UV-spectrophotometer. The resulted absorbance was mathematically converted into a drug concentration. %EE and %DL were calculated by the given below equation:

$$\%\;\mathrm{EE}=\frac{\mathrm{Total}\;\mathrm{BF}-\mathrm{Unentrapped}\;\mathrm{BF}}{\mathrm{Total}\;\mathrm{BF}}\times 100$$

$$\%\;\mathrm{DL}=\frac{\mathrm{Total}\;\mathrm{BF}-\mathrm{Unentrapped}\;\mathrm{BF}}{\mathrm{Weight}\;\mathrm{of}\;\mathrm{NLCs}}\times 100$$

#### 2.2.8. Formulation of BF-NLCs Gel

The point prediction-based optimized BF-NLCopt was converted into a gel by using Carbopol 934 as a gelling agent. Carbopol (0.75, 1, 1.25% *w*/*v*) was dispersed in distilled water overnight, and then the prepared BF-NLCopt dispersion was added with continuous stirring to form the uniformed dispersion. Finally, the triethanolamine was added to the gel system for neutralization of pH.

#### 2.2.9. Gel Evaluation

The prepared BF-NLCopt gel (10 mg) was taken and dissolved in 10 mL of methanol. The sample was centrifuged at 15,000 rpm for 30 min (Cooling centrifuge, Remi Lab, Mumbai, India). The supernatant was collected, filtered, diluted, and the absorbance was measured by UV-spectrophotometer. The absorbance was mathematically converted into the amount of drug present in the formulation. The viscosity of the gel sample was evaluated by Brookfield viscometer at room temperature. The viscosity was measured by using spindle number 6 at 15 rpm [24]. The pH was measured by dispersing 100 mg of gel into distilled water (10 mL) using a digital pH meter (AS218, Eutech Instrument, Singapore) [25]. The measurement of gel spreadability is an important parameter to check the flow of gel. The measured quantity of BF-NLCopt gel (0.5 g) was placed on the glass slide within the pre-marked diameter of 1 cm and covered with another slide. The weight (500 g) was placed over the slide for 10 min, and the increase in diameter due to the spread of gel was noted [26]. The extrudability test was carried out to assess the extrusion of gel from a collapsible tube on the application of constant weight. The collapsible tube containing BF-NLCopt gel (20 g) was pressed by applying a constant load of 1000 gm at the crimp end. The extruded gel was collected after opening the cap and weighed.

#### 2.2.10. In Vitro Release Study

The in vitro release study of BF-NLCopt gel and BF-NLCopt was determined by dialysis membrane (MW12,000 kDa, Sigma Aldrich, USA). The prepared formulations (BF-NLC gel and BF-NLCopt) containing ~5 mg BF were filled into a pretreated dialysis bag (1.0 cm diameter) and both ends were tightly tied. The dialysis bag was dipped into a beaker containing release media (500 mL, phosphate buffer, pH 6.8). The temperature was maintained at 37 ± 0.5 °C throughout the study with regular stirring (50 rpm). The released content (5 mL) from each sample was withdrawn at a specified time and replaced with the same volume. The sample was filtered, diluted with the same medium and the drug concentration at each time point was measured by UV-spectrophotometer [1]. The drug release was calculated and the graph between time vs.% drug release was plotted to calculate the maximum BF release. The release study was performed in triplicate and the release data of BF-NLCopt gel was applied into various release kinetic models to identify the release mechanism. The data were fitted into zero-order, first-order, Higuchi, Korsmeyer Peppas, and Hixson-Crowell model, and the best fit was selected based on the maximum regression (R^2^) value.

#### 2.2.11. In Vitro Permeation Study

The eggshell membrane was used to evaluate the prepared formulations for the permeation study because it is similar to the stratum corneum of human skin [27,28]. The eggs were procured from a poultry house and dipped into concentrated 0.1*N* HCl. The shell was removed, the membrane was collected and cleaned with distilled water. The membrane was mounted between the donor and acceptor compartment of the diffusion cell (area 1.5 cm^2^). The diffusion media (10 mL) phosphate buffer (pH-6.8) were filled in the receptor compartment. The temperature was maintained at 37 ± 0.5 °C throughout the study. The formulations (BF-NLCopt, BF-NLCopt gel, BF-gel containing equivalent to 2 mg of BF) were filled into the donor compartment. At a predetermined time interval, 2 mL of sample was withdrawn and replaced with the same volume to maintain the uniformity in volume. The collected sample was filtered through a membrane filter (0.45 µm) and absorbance was measured by the reported HPLC method to calculate the amount of BF permeated [29].

#### 2.2.12. Antifungal Activity

The antifungal sensitivity activity was analyzed by cup plate diffusion method using Sabouraud’s dextrose agar medium following a previously reported study [30]. The fungal broth culture *(Candida albicans* and *Aspergillus fumigatus*) was standardized in the growth medium. The required quantity medium was prepared and transferred in a clean sterilized Petri plate at 121 °C (15 PSI). The plates were kept aside for 15 min to solidify the media. The wells (6 mm) were prepared using a sterilized stainless-steel borer in the sterilized condition. The samples BF-NLCs-gel and BF-gel were placed into the wells. The plates were kept for 2 h at room temperature to diffuse the sample into the medium and then transferred into the incubator. The zone of inhibition (mm) (ZOI) was measured at 12 h and 24 h for each test sample and compared with the standard.

#### 2.2.13. Irritation Study

The irritation study of BF-NLCs-opt-gel was done by using chick chorioallantoic membrane (CAM) [31]. The irritancy of a developed gel was measured by measuring its ability to induce toxicity in the CAM. The fertilized hen eggs were collected from a poultry farm and incubated into a humidity incubator (Komeg, Tech industrial Ltd., Shenzhen, Guangdong, China) for ten days at 37 ± 0.5 °C and 55 ± 2% RH. The eggs were manually rotated every 24 h. On the 10th day, the eggs were removed from the incubator and the outer shell of the eggs was removed from the air chamber side with the help of forceps. Sterilized normal saline was added for clear visibility of CAM. The formulations (BF-NLCs-opt-gel), normal saline (negative control), and sodium lauryl sulphate (1% *w*/*v*, positive control) were added over CAM for evaluation of irritation. The irritation score was noted at a definite time interval (0.5, 2, and 5 min) and compared to the standard irritation score. No visible hemorrhage score 0–0.9 (non-irritant), membrane decolorization score 1–4.9 (mild irritant), hemorrhage and structured partially covered score 5–8.9 (moderate irritant), hemorrhage and structure covered completely score 9–20 (severe irritant) were seen [32].

#### 2.2.14. Statistical Analysis

All experimental study data were expressed in mean ± SD (*n* = 3). The GraphPad Prism software (San Diego, CA, USA) was applied for statistics. One-way ANOVA was employed followed by the Tukey–Kramer multiple comparison test to analyze statistically significant different samples and control and *p* < 0.05 was taken for statistical significance.

## 3. Result and Discussion

### 3.1. Screening of Lipids and Surfactant

**Figure 1:**
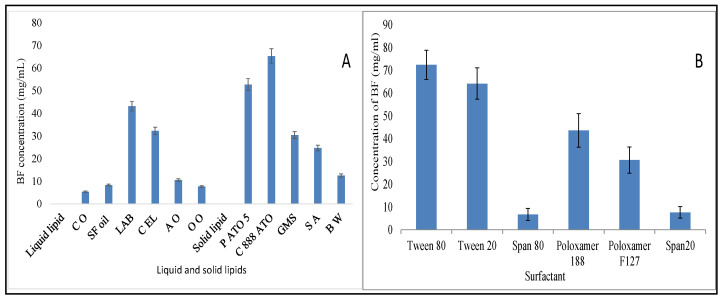
(**A**): Solubility study result of BF in different liquid and solid lipids. Each study performed in triplicate and data shown as mean ± SD. (**B**): Solubility study result of BF in different surfactants. Each study performed in triplicate and data shown as mean ± SD.

The solubility profile of the drug in solid, liquid lipid, and surfactant is shown in Figure 1A,B. The criterion of each component selection was the maximum solubility of the drug. The order of solubility of BF in various lipids is compritol 888 ATO > precirol ATO 5 > glyceryl monostearate > stearic acid > beeswax. The highest solubility of BF was found in compritol 888 ATO (65.24 ± 7.48 mg/mL). The order of solubility of BF in liquid lipid is in the order of labrasol > cremophore EL > almond oil > sunflower oil > olive oil > castor oil (Figure 1A). The highest solubility of BF was found in labrasol (43.12 ± 6.43 mg/mL). The tested surfactant depicted the BF solubility in order of tween 80 > tween 20 > poloxamer 188 > poloxamer F127 > span 20 > span 80 (Figure 1B). Tween 80 showed the maximum solubility of BF (72.45 ± 6.45 mg/mL). So, from the preliminary solubility study, compritol 888 ATO, labrasol, and tween 80 were selected as solid lipid, liquid lipid, and surfactant for the preparation of NLCs.

### 3.2. Miscibility of Solid and Liquid Lipid

The selected solid and liquid lipid were mixed at different proportions to select the optimized blend to use in the preparation. Among the tested ratios, 6:4 was found to be the best composition. This ratio showed no phase separation, as well as no oil droplet was found after smear formation in the filter paper, so the ratio 6:4 was used for further study in the preparation of BF-NLCs.

### 3.3. Optimization

BF-NLCs were optimized using a three-factor three-level Box Behnken formulation design approach to select the optimum composition with minimum particle size and high entrapment efficiency. The design showed 17 formulation compositions with five common compositions to check the error in the results [21,22]. The result of particle size (Y_1_) and entrapment efficiency (Y_2_) was added to the software to interpret the results. The software generated predicted values, polynomial equations, contour, and 3D response surface graphs to evaluate the interaction of independent factors on dependent factors. The actual particle size and entrapment efficiency were compared to the predicted value generated by the software and the result was found to a closer value (Table 2). The summary of regression analysis for the particle size and entrapment efficiency is depicted in Table 3. For all responses, the best fit model was found to be quadratic with the highest value of correlation coefficient (R^2^). The quadratic response is ideal for the optimization because the used independent variable showed individual as well as a combined effect on the dependent variables. The predicted R^2^ was found to be in good agreement with the adjusted R^2^ for both responses. ANOVA value was found to be significant and the F value showed a value greater than 4. Lack of fit was found to be non-significant and residual values results support the optimization (Table 4). The calculation of percentage prediction was performed to determine the accuracy of the software. The negative sign in the polynomial equation corresponds to an opposite relationship and the positive sign favors the interaction of independent variables with the dependent variables. The overall combined desirability was found to be closer to unity, which confirms that the tested independent variable was found suitable for the optimization.

### 3.4. Effect of Independent Variables on Particle Size (Y_1_)

**Figure 2:**
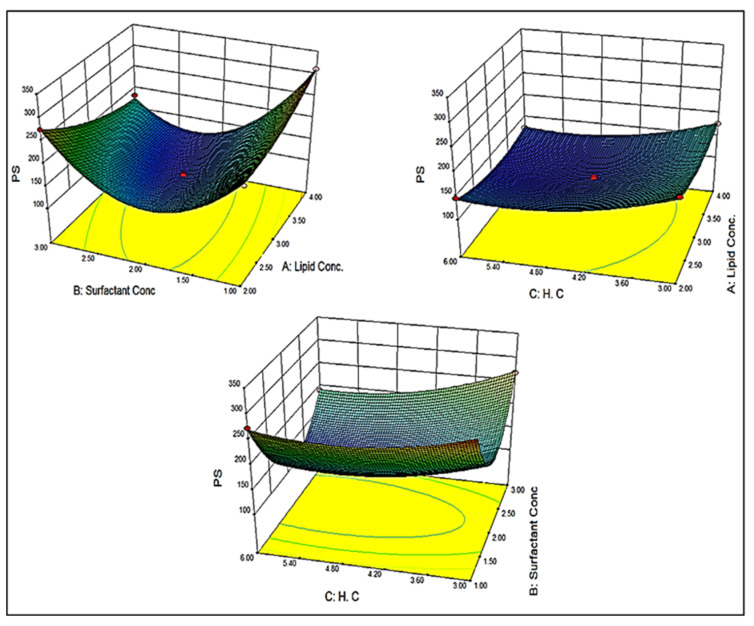
3D response surface plot showing the effect of independent variables on the particle size.

The particle size of different batches was found to be in the range of 113.47 (BF11) to 311.75 nm (BF8). A significant difference in particle size was observed due to the variation in the composition of lipid (A), surfactant (B), and homogenization cycle (C). The polynomial Equation (1) and 3D response plot (Figure 2) showed the effect of independent variables on particle size:

Y_1_ = +117.81 + 8.59A − 26.51B − 21.65C − 41.19AB + 0.48AC − 12.36BC + 19.04A^2^ + 116.02B^2^+ 25.14C^2^ (1)

Lipid showed a positive effect on particle size. So, the optimum concentration of lipid is necessary to obtain the minimum size. The particle size increases with an increase in the lipid concentration due to the availability of higher concentration lipid. This might be due to the lack of sufficient surfactant which solubilizes the drug and reduces the interfacial tension. At a low concentration of surfactant, the aggregation of particles takes place and the particle size increased [33]. The surfactant (B) showed the oppositive effect on the particle size. It influences particle size by giving stability [23]. As the concentration of surfactant increased, the particle size decreases. The surfactant reduces the available space to accommodate the drug [20]. Like surfactants, the homogenization cycle (C) also showed a negative effect on particle size. As the homogenization cycle increases, the particle size decreases due to the generation of high force which is responsible for the breaking and hence reducing the particle size.

Equation (1) also showed the combined effect of various variables on the particle size. The combination of lipid (A) and surfactant (B) showed a negative effect on particle size. So, from the equation, we can say that surfactant (B) has a more prominent effect on particle size than lipid (A). In the case of factors lipid (A) and homogenization cycle (C), it showed a positive effect. As the concentration of lipid and homogenization cycle increases the particle size also increases. At a high homogenization cycle, the lipid may form an aggregate due to the lack of sufficient surfactant (B) concentration. The combined effect of factors surfactant (B) and homogenization cycle (C) showed a negative effect on the size. The combined effect was also found to be similar to the individual effect of surfactant (B) and homogenization cycle (C).

### 3.5. Effect of Independent Variables on Entrapment Efficiency (Y_2_)

The entrapment efficiency of different compositions was found to be in the range of 66.74 (BF1) to 87.42% (BF9). A significant difference in entrapment efficiency was observed due to the variation in the composition of lipid (A), surfactant (B), and homogenization cycle (C). The polynomial Equation (2) concern to entrapment efficiency is given below:

Y_2_ = +84.85 + 3.03A − 2.069B − 1.01C + 0.61AB + 0.030AC − 1.93BC − 3.28A^2^ − 8.69B^2^ − 1.50 C^2^ (2)

**Figure 3:**
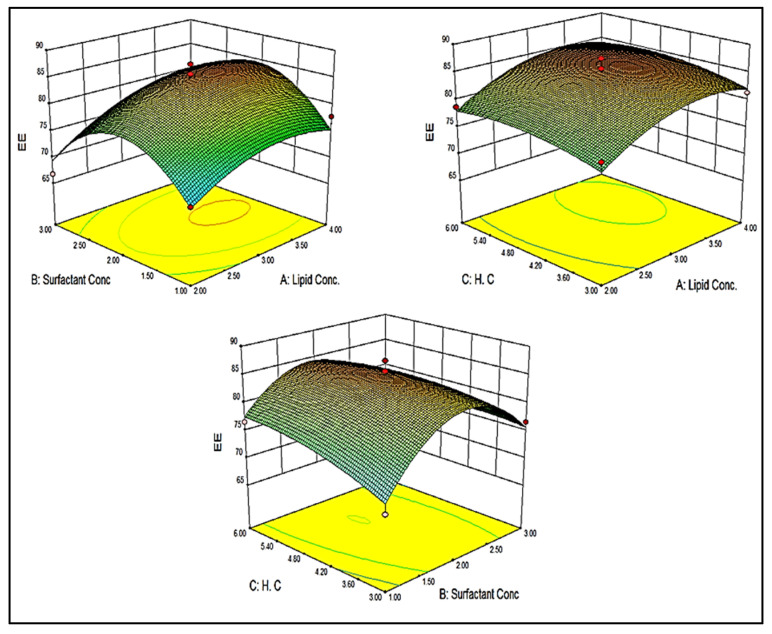
3D response surface plot showing the effect of independent variables on entrapment efficiency.

The polynomial equation and 3D response plot (Figure 3) showed a significant effect of lipid (A), surfactant (B), and homogenization cycle (C) on entrapment efficiency. The increase in the concentration of lipid (A) leads to an increase in the BF entrapment in the NLCs. The use of lipid blends (solid and liquid lipid) gives more space to accommodate more drugs. From the solubility study data, the BF showed good solubility in solid as well liquid lipid, so more amount of drug solubilize and gives greater entrapment. The second-factor surfactant (B) showed an overall negative effect on entrapment efficiency. An increase in surfactant concentration decreases the entrapment efficiency. At the initial stage, with the increase in surfactant concentration, the entrapment efficiency was increased; however, with further increase, there was a measurable reduction in entrapment efficiency, which might be due to drug leakage into the external environment (BF1 70.59% and BF3 66.74%) [20,34]. At a high concentration of surfactant, the drug may leach out from the lipid blend. The homogenization cycle (C) also depicted the negative effect on entrapment efficiency. At a higher homogenization cycle, the high shear force is generated which is responsible for the breakdown of particles and hence leaching of drug from lipid nanoparticles takes place and reduces the drug entrapment. The combination of lipid (A) and surfactant (B) showed a positive effect on drug entrapment. So, from the equation, we can say that surfactant (B) has a less prominent effect than the lipid (A). In the case of factor lipid (A) and homogenization cycle (C), it showed a positive effect. As the concentration of lipid and homogenization cycle increases the entrapment also increases. At a high lipid concentration and homogenization cycle, the amount of lipid breakdown is higher and a higher amount of BF is entrapped. The combined effect of factors surfactant (B) and homogenization cycle (C) showed a negative effect on the size. The combined effect was also found to be similar to the individual effect of surfactant (B) and homogenization cycle (C).

### 3.6. Point Prediction

**Figure 4:**
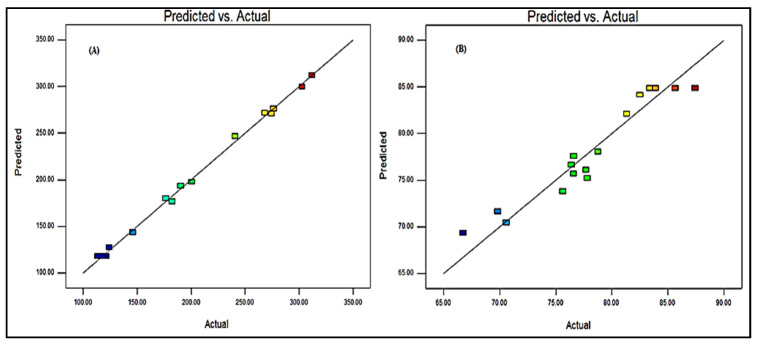
Actual and predicted value of particle size (**A**) and entrapment efficiency (**B**).

The formulation was further optimized by the software-generated point prediction method. The point prediction was made by making minute changes in the value of independent parameters to get the more close and accurate value of these parameters and their effect on the responses. The main objective of this step to get more accurate results and validate the resulted model. The optimized formulation (BF-NLCopt) shows the composition of lipid concentration 2.6%, surfactant concentration 2.4%, and the homogenization cycle of 4. This composition showed a particle size of 119.29 nm and an entrapment efficiency of 86.35%. The predicted value was found to be very closer to the practical value. The predicted particle size was found to be 112.14 nm with an entrapment efficiency of 88.56%. There was a non-significant difference in the actual and predicted particle size, and entrapment efficiency was observed (Figure 4). It indicates that the model was well fitted between them.

### 3.7. Particle Characterization

**Figure 5:**
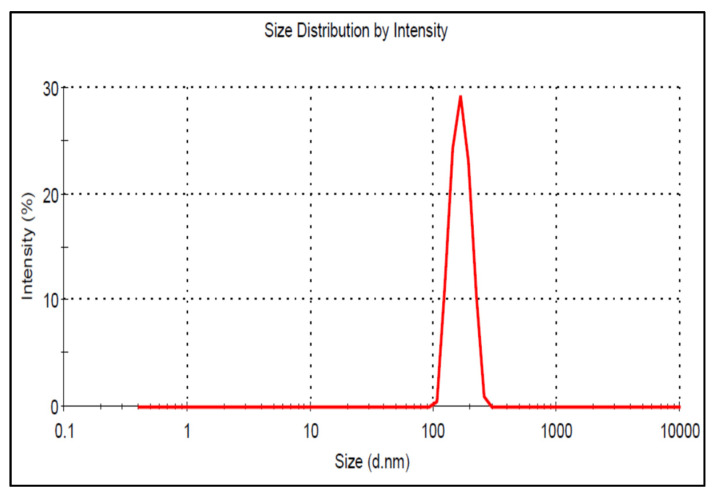
Particle size image of BF-NLCopt.

**Figure 6:**
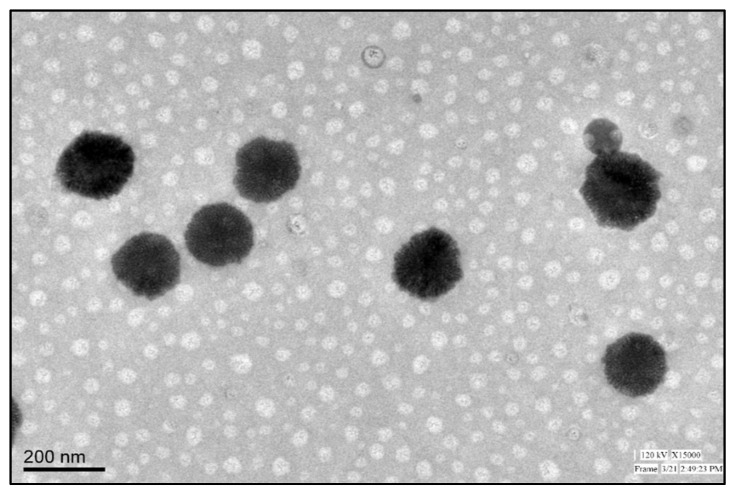
Surface morphology image of BF-NLCopt.

The particle size of the prepared BF-NLCs formulations was analyzed and is given in Table 2. The particle size of BF-NLCs was found in the range of 113.47 (BF11) to 311.75 nm (BF8). The selected formulation BF-NLCopt showed a particle size of 119.29 ± 3.23 nm (Figure 5), PDI of 0.29 with a ZP of −28 mV. The low PDI (<0.5) and zeta potential value (−28 mV near to optimum, ±30 mV) support the physical stability of the prepared formulation. The particle size plays an important role in the permeation of the drug through the skin and helps in the retention of the drug in the epidermis. Particle size more than 100 nm will not penetrate the lower skin layers. Nanoparticles (>100 nm) preferentially permeate into the follicles, but not the dermis, enabling high accumulation within the follicular reservoir. The epidermis releases the drug for a prolonged duration to treat the topical fungal infection [35,36]. As previously reported that lipid nanoparticles lower than 100 nm were capable to penetrate the deeper layer of skin and bigger size nanoparticles were incapable to do so [37]. Moreover, NLCs provide greater drug permeation and therapeutic efficacy by offering close contact with the stratum corneum. Due to the lipid, NLCs have bioadhesion properties and form an occlusive film which decreases the water loss from the transepidermal layer. NLCs also facilitate the penetration of the drug into deeper skin [19]. The morphology of BF-NLCs-opt was found to be spherical and is given in Figure 6.

### 3.8. Entrapment Efficiency

The entrapment efficiency of the prepared BF-NLCs was determined by the indirect method and the data are given in Table 2. The maximum entrapment efficiency was found in the formulation BF1 (66.74%) to BF7 (87.42%). The selected optimized formulation BF-NLCopt showed an entrapment efficiency of 86.35 ± 4.11%. The selected formulation showed a drug load of 11.21 ± 0.67%. NLCs were prepared with a blend of solid lipid and liquid lipid. The solid lipid encloses the oil droplets of liquid lipid which solubilizes the greater amount of drug and helps to get high entrapment and loading. The presence of liquid lipid in the NLCs affects the loading and entrapment by creating imperfections in the order crystal structure and gives more space to accommodate a greater amount of drug [23].

### 3.9. Formulation of BF-NLCopt Gel

Due to the low viscosity of NLCs dispersion, it displays unsuitable rheological properties so it is difficult to apply to the skin layer. To overcome these issues, the low viscosity dispersion system was converted into a hydrogel system to provide precise spatial and temporal control of active ingredient release. Carbopol was selected as the gelling agent due to its compatibility with nanoparticulate delivery, easy formulation, thermal stability, optimum rheological properties. The use of triethanolamine (organic base) as neutralizing agent improves the stability of lipid nanoparticles [38]. BF-NLCs-opt was successfully transformed into gel using Carbopol in three different concentration (0.75, 1, 1.25% *w*/*v*) to select the optimum concentration. Among the three concentrations, BF-NLCopt gel prepared with Carbopol (1% *w*/*v*) showed good spreadability for the gel formulation. The other tested concentration (0.75 and 1.25%) results do not show a satisfactory result.

### 3.10. BF-NLCs Gel Characterization

The drug content indicates the % of BF existing in the gel formulation. It is the ratio (%) of the drug present in formulation to the actual amount of drug taken for gel formulation development. The high drug content is a desirable character of pharmaceutical formulation. Here, the drug content of the prepared BF-NLCopt gel was found to be 99.45 ± 2.11%. The high drug content approved the authenticity of the adopted method to develop the gel formulation. The pH of BF-NLCopt gel was determined by pH meter at room temperature and the value was found to be 6.5 ± 0.2. The pH was found to be within the limit of topical gel and does not produce any toxicity [7]. The viscosity of BF-NLCopt gel was evaluated by Brookfield viscometer and the result was found to be 519 ± 1.43 CPs. The value of viscosity is significantly affected by the particle size and PDI. The higher value gives a more viscous formulation [39]. In this formulation, the particle size and PDI value both are lower and support the findings. Generally, the viscosity of gel formulations reflects consistency. The viscosity decreases with increasing the rate of shear rate (shear thinning, Non-Newtonian flow) in the case of gel formulation. The viscosity must be optimum to show better adherence to the skin and spreadability [36,40]. Spreadability is a very important parameter for the determination of the spreading capacity of gel on the skin. The good spreadability of topical formulation gives better application to inflamed or diseased skin. It was determined by application of weight and expressed as the area of spread of the gel. The area of the spread gel was found to be 6.35 ± 0.56 cm in low spreading time (1.5 s, visually observed). The viscosity decreases when applied with shear (might be due to the characteristic pseudoplastic behavior of gel), which confirm the characteristic of high spreadability due to the decrease in viscosity when applying a certain force. Moreover, the gel retained the property of remaining at the application site without draining, indicating the adherence ability of the developed gel formulation.

### 3.11. In Vitro Release Study

**Figure 7:**
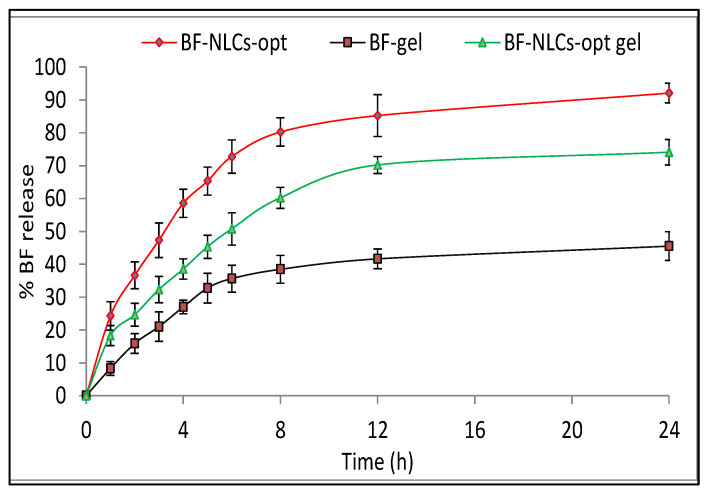
In vitro release study profile of BF-NLCopt, BF-NLCopt gel, and BF-gel. Each study was performed in triplicate and data are shown as mean ± SD. Comparison of permeation study of BF gel with BF-NLCsopt gel and BF-NLCs-opt. The significant differences observed at *p* < 0.001.

The in vitro drug release study of BF-NLCopt, BF-NLCopt gel, and BF-gel was done by using a dialysis membrane. BF-NLCopt, BF-NLCopt gel, and BF-gel showed the cumulative BF release of 88.09 ± 3.01%, 65.09 ± 4.37%, and 34.54 ± 3.87% in 24 h, respectively (Figure 7). BF-NLCopt gel showed a significant (*p <* 0.05) low release of BF than BF-NLCopt dispersion due to the presence of Carbopol gel matrix. It hindered the release of BF from the gel matrix and slowly released due to the extra barrier. BF-NLCopt gel exhibited a significant (*p <* 0.05) higher release of BF than BF gel (conventional). The presence of lipid and surfactant in BF-NLCopt gel reduces the surface interfacial tension, which enhances the solubility and dissolution of BF. BF-NLCopt and BF-NLCopt gel exhibited initial burst release due to the diffusion of unentrapped BF quickly to release media. Later, sustained BF release was observed from the lipids (solid and liquid lipid) for BF-NLCs-opt and lipid as well as Carbopol matrix for BF-NLCopt gel. These release patterns agreed with the previous research study reported by [19,26]. The biphasic release behavior is ideal for the topical formulation because the burst release gives the amount of drug at the target site to achieve therapeutic concentration and the slow release helps to maintain the therapeutic concentration [37,41]. The release data fitted to the different kinetic models, i.e., zero-order, first-order, Higuchi, Korsmeyer-Peppas, Hixson-Crowell. The R^2^ value for each model was found to be 0.7399 (zero-order), 0.834 (First order), 0.892 (Higuchi), 0.9506 (Korsmeyer-Peppas), and Hixson-Crowell (0.897). The maximum R^2^ (0.9506) value was found to be for Korsmeyer-Peppas, so this model was selected as the best kinetic release model. The *n* value was found to be 0.48 (<0.5), indicating the release of the drug by diffusion (Fickain type) [42].

### 3.12. Permeation Study

**Figure 8:**
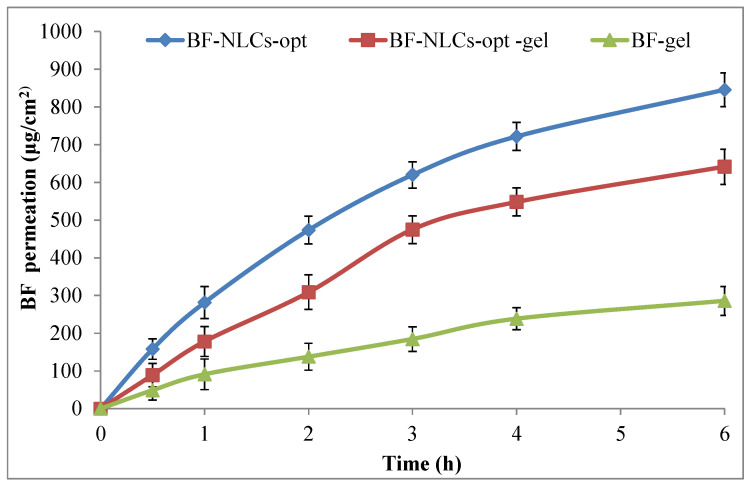
Permeation study profile of BF-NLCopt, BF-NLCopt gel, and BF-gel. Each study was performed in triplicate and data shown as mean ± SD. Comparison of permeation study of BF gel with BF-NLCsopt gel and BF-NLCs-opt. The significant differences observed at *p* < 0.001.

The in vitro permeation study of BF-NLCopt, BF-NLCopt-gel, BF-gel was performed by diffusion shell using an eggshell membrane and data are shown in Figure 8. The amount of BF permeated from BF-NLCs-opt, BF-NLCs-opt-gel, and BF-gel through the membrane was found to be 845.52 ± 44.59 µg/cm^2^ (42.28 ± 2.22%), 641.37 ± 46.59 µg/m^2^ (32.07 ± 2.32%), and 285.52 ± 38.59 µg/cm^2^ (14.28 ± 1.92%). There was a highly significant (*p <* 0.001) enhancement in the BF permeation from BF-NLCs-opt and BF-NLCsopt gel than BF gel. The greater permeation was found due to the nanometric size and presence of surfactant and lipid which act as permeation enhancers. The presence of surfactant helps solubilize the drug as well as helps open the pores of the membrane. In addition, There was a significant (*p <* 0.001) difference in the permeation between BF-NLCs-opt and BF-NLCopt gel. A lesser amount permeated from the gel formulation due to the slow diffusion of BF from the Carbopol gel matrix. The slow permeation from BF-NLCopt gel is ideal for topical preparation. The drug slowly permeates the skin and retains on the skin for a greater period. The presence of the drug at the target area gives a more prominent effect than the prepared NLCs formulation.

### 3.13. Anti-Fungal Study

**Figure 9:**
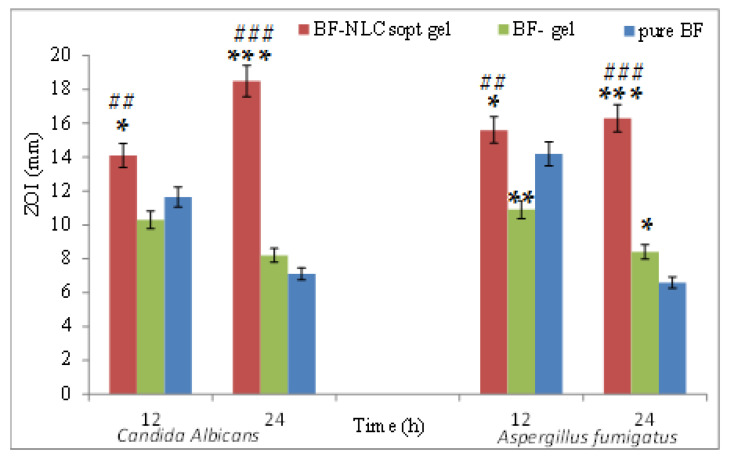
Antifungal study profile of Butenafine NLCopt gel, Butenafine gel, and Butenafine dispersion. Each study was performed in triplicate and data are shown as mean ± SD. * Comparison of antifungal activity of pure BF with BF-NLCsopt gel and BF-gel, Comparison of antifungal activity of BF-gel with BF-NLCsopt gel. *, **, ***, ##, ### show the significant differences at *p* < 0.05, *p* < 0.01, *p* < 0.001; respectively.

The anti-fungal activity of BF-NLCopt gel, BF-gel, and BF dispersion was analyzed on *Candida albicans*, and *Aspergillus fumigatus* by agar diffusion method and data are shown in Figure 9. BF-NLCopt gel showed a ZOI of 14.1 ± 1.6 mm and 18.5 ± 2.1 mm against *Candida albicans*, whereas 15.6 ± 2.1 mm and 16.3 ± 1.6 mm against *Aspergillus fumigatus* in 12 h and 24 h, respectively. BF-gel showed ZOI of 10.3 ± 0.8 mm and 8.2 ± 0.9 mm against *Candida albicans* at 12 h and 24 h. Whereas, the observed ZOI was found to be 10.9 ± 1.1 mm and 7.1 ± 1.2 mm against *Aspergillus fumigatus* at the same time point, respectively. BF-dispersion showed good ZOI at 12 h (11.6 ± 1.3 mm) and very low ZOI at 24 h (6.9 ± 0.9 mm) against *Candida albicans.* In the case of *Aspergillus fumigatus*, BF dispersion showed a similar result as high ZOI at 12 h (14.1 ± 1.4 mm) and low ZOI at 24 h (6.6 ± 0.7 mm). There was a significant (*p* < 0.05) difference observed between the evaluated ZOI between BF-NLCopt gel and BF gel and BF dispersion. BF-NLCopt gel showed a marked enhancement in the ZOI at both time points. The higher activity is due to the higher solubility of BF in the used lipids and surfactant; and higher concentration of BF is available at the target area. The nanometric-sized BF-NLC particles slowly diffuse and produce antifungal activity.

### 3.14. Irritation Study

The irritation study was performed by HET- CAM using the egg membrane and the irritation score was calculated. The experiments were observed for any sign of lysis, hemorrhage, coagulation of blood vessels for a maximum duration of 5 min. A test is considered acceptable if the negative and positive controls each induce a response that falls within the classification of nonirritating and severely irritating, respectively. The comparison of the study was performed between the positive control (1% *w*/*v*, SLS), negative control (0.9% NaCl), and BF-NLCopt-gel. The positive control-treated CAM showed a cumulative score of 17 (indicates severe irritation). The negative control-treated chorioallantoic membrane showed a cumulative score of 0, which is considered as non-irritant. BF-NLCopt-gel-treated CAM membrane exhibited a cumulative score of 0.17 with no signs of lysis, hemorrhage, coagulation of blood vessels after 5 min indicating that the optimized gel formulation was nonirritant and nontoxic. The overall score of the study of BF-NLCopt gel showed a score between 0 and 0.9 considered as non-irritant and safe. The finding of the study agreed with previously published research work [43].

## 4. Conclusions

The prepared BF-NLCs were optimized using three factors at three-level Box Behnken design. The optimized formulation was converted to carbopol gel to enhance skin contact. The prepared formulations showed nanometric size with high drug entrapment efficiency. The results showed an enhanced drug release pattern from BF-NLCopt than BF-NLCopt-gel. The permeation data showed higher permeation from BF-NLCopt and BF-NLCopt-gel across the tested membrane. Furthermore, the formulation showed optimum viscosity, pH, and spreadability. The irritation and antifungal result studies showed no irritation to the tested CAM as well as BF-NLCopt gel showed a marked enhancement in the ZOI at both time points. The overall study revealed that the butenafine nano lipid carrier-based gel system acts as a potential delivery system in the treatment of anti-fungal disease.

## Figures and Tables

**Table 1:** Formulation variables range used to optimize BF-loaded NLCs.

Parameters	Constraints		
	Lower Value	Medium Value	Upper Value
Independent variables			
Lipid concentration (A, %)	2	3	4
Surfactant concentration (B, %)	1	2	3
Homogenization cycle (C, n)	2	4	6
Dependent variables	Goals		
Particle size (Y_1_, nm)	Minimize		
Entrapment efficiency (Y_2_, %)	Maximize		

**Table 2:** Composition of BF-loaded NLCs with their actual and predicted particle size and encapsulation efficiency.

Batch No.	Lipid (A, %)	Surfactant (B, %)	Homogenization Cycle (C, n)	Particle Size (nm) (Y_1_)		Entrapment Efficiency (%) (Y_2_)	
				Actual	Predicted	Actual	Predicted
BF1	2	3	4	182.55	179.48	66.74	69.31
BF2	3	1	2	302.77	299.76	69.82	71.65
BF3	4	3	4	276.36	276.57	76.44	76.60
BF4	3	2	4	118.14	117.81	83.91	84.85
BF5	2	2	6	146.23	143.44	77.76	78.02
BF6	3	2	4	118.37	117.81	83.38	84.85
BF7	4	2	6	124.25	127.23	82.51	84.15
BF8	4	1	4	311.75	311.97	77.81	75.24
BF9	3	2	4	117.85	117.81	84.42	83.85
BF10	3	3	6	190.44	193.45	75.65	73.82
BF11	3	2	4	119.47	117.81	85.65	84.85
BF12	3	3	2	268.28	271.18	76.59	75.66
BF13	2	2	2	200.69	197.71	78.70	76.06
BF14	3	2	4	117.21	117.81	83.89	84.85
BF15	3	1	6	274.38	276.14	76.61	77.54
BF16	2	1	4	240.78	246.76	70.59	70.39
BF17	4	2	2	176.78	179.57	81.33	82.07

**Table 3:** Statistical model summary of regression analysis results for response Y_1_, and Y_2._

Model	R^2^	Adjusted R^2^	Predicted R^2^	SD	% CV	Remark
Particle size (Y_1_)						
Linear	0.9147	0.8155	0.8465	7.14		
2F1	0.9315	0.9295	0.9012	8.02		
Quadratic	0.9979	0.9951	0.9718	5.04	2.61	Suggested
Entrapment efficiency (Y_2_)						
Linear	0.9031	0.8423	0.8391	5.90		
2F1	0.9183	0.8305	0.8026	6.60		
Quadratic	0.9962	0.9312	0.9215	2.37	3.02	Suggested

**Table 4:** ANOVA of quadratic model for responses of developed BF-NLCs.

ANOVA Results	Particle Size (Y_1,_ nm)	Entrapment Efficiency (%) (Y_2_)
Regression		
Some of square	83149.71	494.61
Degree of freedom	9	9
Mean square	9238.86	54.96
F-value	363.48	9.75
P	<0.0001	<0.0033
Influence	Significant	Significant
Lack of fit-test		
Some of square	143.43	28.23
Degree of freedom	3	3
Mean square	47.81	9.51
F-value	5.54	3.36
P	0.0658	0.1363
Influence	Non-significant	Non-significant
Residual		
Some of square	177.93	39.44
Degree of freedom	7	7
Mean square	25.42	5.63

## Data Availability

Not applicable.
